# Managing Ifosfamide-Induced Arginine Vasopressin Resistance: Diagnostic and Treatment Strategies

**DOI:** 10.7759/cureus.81236

**Published:** 2025-03-26

**Authors:** Avia A Williams, Arslan I Pir Muhammad, Rupam Ruchi, Rozina Ali

**Affiliations:** 1 Internal Medicine, Cornwall Regional Hospital, Montego Bay, JAM; 2 Medicine, Ziauddin University, Karachi, PAK; 3 Nephrology, Hypertension, and Transplantation, University of Florida-College of Medicine, Gainesville, USA

**Keywords:** acute kidney injury, arginine vasopressin deficiency, arginine vasopressin disorder, arginine vasopressin resistance, diabetes inspidus, drug induced fanconi syndrome, hypernatremia, ifosfamide, polyuria, proximal tubulopathy

## Abstract

The early recognition of ifosfamide-related renal dysfunction, including arginine vasopressin resistance (previously referred to as diabetes insipidus), which is characterized by polyuria due to the inability of the collecting duct to concentrate urine, as well as direct proximal tubular injury, which is characterized by numerous metabolic disturbances including hypophosphatemia, metabolic acidosis, hypokalemia, and glucosuria, is paramount to timely initiation of treatment and titration of desmopressin. In this report, we present a case of a 42-year-old female with a history of spindle cell carcinoma of the left thigh, who was referred to the inpatient nephrology consult service for acute kidney injury, polyuria, and metabolic derangements following her sixth cycle of doxorubicin-ifosfamide chemotherapy. The patient was treated with supratherapeutic doses of desmopressin, with successful improvement of her polyuria. We review the pathophysiology of different forms of ifosfamide-associated nephrotoxicity, including arginine vasopressin resistance, the challenges of diagnosing arginine vasopressin disorders, and the utility of desmopressin in the management of arginine vasopressin resistance. This case also highlights the clinical implications of using copeptin in the diagnosis of arginine vasopressin resistance, leading to effective treatment and improving patient outcomes.

## Introduction

Several frequently used chemotherapeutic drugs have the potential to cause renal toxicity, and ifosfamide is one such common agent with known varying nephrotoxic risks. A well-known risk is the impairment of the reabsorption function of the proximal convoluted tubule, leading to Fanconi syndrome [1]. Prominent findings of Fanconi syndrome are renal tubular acidosis due to the impaired proximal reabsorption of bicarbonate, in addition to phosphaturia, glycosuria, aminoaciduria, and hyperuricosuria [2]. The lesser-known toxicity is the development of tubulointerstitial injury resulting in chronic kidney disease, even years after initial exposure to the drug [3]. Additionally, ifosfamide can also cause arginine vasopressin resistance, which is a hereditary or acquired condition characterized by polyuria due to the inability of the collecting duct to concentrate urine. Arginine vasopressin (AVP) is the antidiuretic hormone that acts on the V2 receptor in the basolateral membrane of the principal cells of the renal collecting duct to promote free water reabsorption via aquaporin-2 channels, expressed on the apical membrane of the principal cells [4,5]. AVP deficiency, previously referred to as central diabetes insipidus, is a condition also marked by polydipsia and polyuria, owing to the absence of the hormone that participates in concentrating urine. In both arginine vasopressin deficiency and arginine vasopressin resistance, patients experience persistent diuresis, producing high volumes of dilute urine [4].

We report a case of a patient with spindle cell sarcoma of the left thigh, who was treated with combined doxorubicin-Ifosfamide and developed severe hypernatremia and polyuria, alongside findings of acute kidney injury and Fanconi syndrome. Ifosfamide was thought to be the main culprit in her development of arginine vasopressin resistance, and the patient responded favorably to supraphysiological doses of desmopressin (DDAVP). Additionally, we utilized copeptin to aid in the accurate diagnosis of arginine vasopressin resistance. Copeptin, which is co-secreted with arginine vasopressin (AVP), has emerged as a valuable biomarker in the differential diagnosis of arginine vasopressin disorders, particularly in distinguishing between arginine vasopressin deficiency and resistance, as well as primary polydipsia-related polyuria-polydipsia syndromes [6].

## Case presentation

The patient is a 42-year-old female of African ancestry, with a pertinent history of hypertension, diabetes mellitus type 2, and spindle cell sarcoma of the left thigh, diagnosed two years prior. At the time of initial diagnosis, she underwent local definitive tumor resection of her left lateral mid-thigh with negative margins. However, eight months later, she developed local recurrence, with a positive deep margin of the left posterior thigh soft tissue biopsy, as well as metastasis to the left groin region lymph node, requiring neoadjuvant radiation therapy. She was subsequently initiated on doxorubicin-ifosfamide. Her first cycle was administered five months prior to presentation. She was admitted for her sixth cycle of doxorubicin-ifosfamide, but two days following chemotherapy administration, she developed worsening hypernatremia and polyuria. This was associated with altered mental status, prompting transfer to the medical intensive care unit from the general oncology floor.

At the time of the consultation for acute kidney injury, the patient’s hospital medications included apixaban, dexamethasone, docusate sodium, insulin aspart, insulin glargine, lorazepam, rosuvastatin, thiamine, olanzapine, and a multivitamin. She did not have any known drug allergies. At the time of consultation, the patient's physical exam was also remarkable for hypotension, tachycardia, and lethargy. She was also on empiric antibiotics of vancomycin and cefepime for concern for sepsis.

The patient’s sodium on admission was 152 mmol/L (corrected for hyperglycemia, as blood glucose was 444 mg/dL). On day three of her hospitalization, her sodium rose to 173 mmol/L (corrected for hyperglycemia, as blood glucose was 220 mg/dL). On admission, she was also noted to have numerous metabolic derangements and stage 1 acute kidney injury (based on Kidney Disease: Improving Global Outcomes, KDIGO, clinical practice guidelines). The patient’s creatinine on admission was 1.06 mg/dL. Her baseline creatinine was noted to be 0.5 mg/dL based on prior available data. She underwent a computed tomography (CT) scan of the head without contrast and a CT scan with intravenous contrast of the chest, abdomen, and pelvis on the day of transfer to the medical intensive care unit (ICU) as part of an evaluation for altered mental status and acute illness, which was negative for acute pathology. Pertinent lab values on hospital days one, three, five, eight, and 24 are shown in Table 1.

**Table 1:** Available laboratory trends from day one to day 24 of treatment

Parameters	Day 1	Day 3	Day 5	Day 8	Day 24	Reference range
Corrected sodium	152	173	149	145	148	135 - 145 mmol/L
Sodium	145	171	147	145	147	135 - 145 mmol/L
Potassium	3.0	3.3	3.5	3.7	3.3	3.5 - 5.5 mmol/L
Chloride	105	136	128	122	112	98 - 110 mmol/L
Carbon dioxide	25	25	14	19	27	23 - 29 mmol/L
Urea nitrogen	8	8	4	6	8	6 - 20 mg/dL
Creatinine	1.06	0.96	1.04	1.10	0.96	0.8 - 1.2 mg/dL
Glucose	444	220	205	108	145	64 - 100 mg/dL
Phosphorus	-	1.9	2.1	3.2	2.3	2.8 - 4.5 mg/dL
Magnesium	-	2.6	2.2	2.2	1.7	2.9 - 5.3 mg/dL
Serum osmolality	-	338	-	-	-	275 - 295 mOsm/kg
Urine osmolality	-	178	246	410	-	50 - 1100 mOsm/kg
Urine sodium	<10	27	51	60	-	mmol/L

During her hospitalization, the patient was treated for metabolic derangements of hypokalemia and hypophosphatemia with aggressive electrolyte repletion. Phosphorus was found to be 1.9 mg/dL on day two of admission and remained persistently low, <2.5 mg/dL for the majority of her course. Fractional excretion of urea was calculated to be 54.43% (day four) based on the findings of serum uric acid of 2.5 mg/dL, serum creatinine of 1.26 mg/dL, urine uric acid of 10.8 mg/dL, and urine creatinine of 10.0 mg/dL. The patient received multiple boluses of lactated Ringer during the first forty-eight hours of admission due to concern that she had hypovolemic hypernatremia and acute kidney injury.

On day three of her hospitalization, she was initially treated with two one-liter boluses of dextrose 5% in water with a transition to infusion at a rate of 300-600 mL/hr. On day three, a copeptin level was also obtained and returned elevated, at 83 pmol/L. On day four, she was also initiated on intermittent IV desmopressin at a dose of 2 mcg IV Q6hr with a transition to vasopressin infusion, titrated up to 3-6 milli-units/kg/hr. Urine osmolality increased from 178 mOsm/kg to 410 mOsm/kg during this period. On day seven, the patient was started on amiloride as part of the management of arginine vasopressin resistance. However, it was stopped due to volume depletion a few days later. On day 10 of her admission, following seven days of vasopressin infusion, she was subsequently transitioned to oral desmopressin, titrated up steadily from 200 mg by mouth twice daily to 400 mg by mouth three times daily over a subsequent period of seven days. Once her vasopressin regimen was established, and she was determined to be volume repleted, she was started on hydrochlorothiazide (HCTZ) 12.5 mg orally once daily on day 21. She was discharged with instructions to take desmopressin 300 mg orally in the morning and the afternoon, with an additional 400 mg orally nightly. She was also discharged on HCTZ 25 mg orally once daily. The patient’s polyuria was profound. In the first 24 hours of admission, she had 6.3 liters of urine output. On day three of her admission, she had 10.3 liters of urine output. Her highest urine output readings were 13.9 liters on day four and 13.1 liters on day six. After day eight, her polyuria began to improve with recordings of 3-5 liters daily, and she continued to receive careful daily monitoring of volume status and serum electrolyte levels. Her polyuria resolved at the time of discharge.

## Discussion

Ifosfamide, an alkylating oxazaphosphorine used as an anticancer prodrug, has well-documented adverse effects in the pediatric population [2]. Recent research has also examined ifosfamide's impact on adults, particularly focusing on kidney injury. However, more studies are needed to better understand nephrotoxic thresholds with respect to ifosfamide dosing [2,3]. Recent literature suggests that in the adult population, ifosfamide-mediated kidney injury can also lead to tubulointerstitial injury, resulting in progressive chronic kidney disease, even years after the original ifosfamide administration [3]. In our patient’s case, she underwent six cycles of chemotherapy with doxorubicin-ifosfamide.

Ifosfamide-mediated kidney injury results in proximal renal tubulopathy. Although this process is not fully understood, the literature suggests that ifosfamide metabolites like glutathione-depleting chloroacetaldehyde and acrolein accumulate in the proximal renal tubule to promote oxidative stress, causing proximal tubular dysfunction seen in Fanconi syndrome [2,7-9]. Fanconi syndrome is characterized by reduced glomerular filtration and reabsorption, resulting in elevated levels of glucose, amino acids, uric acid, phosphate, and bicarbonate in the urine, ranging from mild to severe [2].

In this patient, urinalysis revealed significant proteinuria and glucosuria. Her fractional excretion of urea was also significantly elevated. Her basic metabolic profile showed severe hypophosphatemia and hypokalemia. Treatment for Fanconi syndrome primarily involves addressing the underlying cause and providing supportive care. For our patient, this meant continuing with replacement therapy to manage Fanconi syndrome symptoms after completing six cycles of doxorubicin-ifosfamide chemotherapy.

Ifosfamide is also associated with arginine vasopressin resistance (AVP-R). Adults can develop AVP-R due to drug toxicity (e.g., lithium), infiltrating lesions (e.g., sarcoidosis), or electrolyte imbalances (e.g., hypercalcemia and hypokalemia) [10]. Unlike congenital AVP-R, which presents in infancy with failure to thrive, acquired AVP-R typically appears later in life with symptoms such as polyuria and/or polydipsia. Laboratory tests for acquired AVP-R usually show hypernatremia and highly dilute urine [4].

Our patient’s symptoms aligned with these findings: sodium levels were as high as 173 mEq/L, and she had significant polyuria with a maximum urine output of approximately 13 liters. Hypokalemia can exacerbate AVP-R by impairing urine concentration, possibly due to decreased aquaporin-2 expression [11]. Our patient was also briefly on amiloride, but it was held due to volume depletion and concern for exacerbating her kidney injury. Thiazide diuretics are commonly used for treating AVP-R. Although the use of thiazide diuretics may seem counterintuitive, the inhibition of the sodium-chloride cotransporter in the distal convoluted tubule induces an increase in the reabsorption of sodium and water in the proximal convoluted tubule, reducing urine output [10]. Our patient was initiated on high doses of desmopressin, initially intravenously, and then transitioned to oral dosing, and responded positively. Her regimen of desmopressin 300 mcg twice daily, with a supplemental dose of 400 mcg nightly, demonstrated consistent improvement and control of her sodium levels. Her response to desmopressin suggested that her AVP-R was due to partial, rather than total, resistance to arginine vasopressin. Our case supports earlier studies that found success with desmopressin as a treatment for arginine vasopressin resistance [11,12].

Copeptin is now recognized as an important biomarker in the differential diagnosis of arginine vasopressin disorders, particularly in distinguishing between arginine vasopressin deficiency and resistance, as well as primary polydipsia-related polyuria-polydipsia syndromes. Recent studies have demonstrated that copeptin levels offered a reliable alternative, correlating well with AVP secretion [6]. A baseline copeptin threshold of >21.4 pmol/L effectively differentiates arginine vasopressin resistance from other etiologies, eliminating the need for water deprivation testing in such cases. In our patient’s case, her copeptin level was elevated at 83 pmol/L. Copeptin is a robust and practical surrogate marker for AVP, streamlining the diagnostic process and improving the accuracy of arginine vasopressin disorder classification. The clinical implications are great, as early detection of AVP-R can lead to early effective treatment.

It is important to note that this patient was started on vasopressin on day three of her hospitalization, despite demonstrating polyuria in the first 24 hours of her admission. Additionally, there may have been a delay in recognizing true hypernatremia, as her serum sodium on day one was 145, prior to correcting for a glucose level of 444. This underscores the importance of evaluating sodium levels concomitantly with glucose levels to avoid missed true hypernatremia. Arginine vasopressin disorder in hospitalized patients can lead to significant morbidity and mortality if not identified early and effectively managed. Persistent polyuria in patients with an arginine vasopressin disorder can lead to severe hypovolemia and electrolyte disturbances that can result in complications such as hypotension, acute kidney injury, and cardiovascular issues, all of which can exacerbate other underlying health conditions and contribute to poor outcomes. In severe cases of untreated arginine vasopressin disorder, patients are at risk of coma, seizures, and, in extreme cases, death. Additionally, persistent symptoms of arginine vasopressin disorder, such as excessive thirst and frequent urination, can impact a patient's overall comfort and quality of life, potentially affecting their ability to participate in rehabilitation and recovery efforts. The recognition of arginine vasopressin disorder early in the hospital course not only improves chances of a faster recovery but also decreases the length of stay, risk of hospital-acquired infections, and complications associated with dysnatremia and hypovolemia. Effective management of arginine vasopressin disorder involves addressing the underlying cause, ensuring adequate fluid and electrolyte balance, and monitoring for complications.

## Conclusions

This case highlights the benefits of diligent early identification of arginine vasopressin resistance to effectively manage the condition and reduce the risk of further acute kidney injury, life-threatening hypernatremia and metabolic derangements, and associated prolonged hospitalization and morbidity risk. Clinical professionals should consider the significant role of desmopressin in treating arginine vasopressin resistance as an active therapeutic approach, alongside close monitoring and maintenance of fluid and electrolyte levels. Additionally, the use of copeptin may be valuable for early detection and support for the diagnosis of arginine vasopressin resistance. For hospitalized patients, multidisciplinary care involving nephrology, endocrinology, and critical care teams may be crucial to optimize outcomes and reduce health complications associated with arginine vasopressin resistance.
